# Therapeutic effect of high-efficiency online hemodiafiltration for recurrent restless legs syndrome in dialysis patients

**DOI:** 10.1007/s10047-020-01164-1

**Published:** 2020-03-30

**Authors:** Kenji Sakurai, Takeshi Saito, Hiromi Hosoya, Yoshitaka Kurihara, Fumi Yamauchi

**Affiliations:** Hashimoto Clinic, 3-21-5 Hashimoto Midori-ku, Sagamihara, Kanagawa 252-0143 Japan

**Keywords:** Hemodiafiltration, α_1_-microglobulin, Restless legs syndrome, Albumin leakage

## Abstract

Two dialysis patients developed recurrent restless legs syndrome. The clinical courses and the association between the α_1_-microglobulin removal rate and the therapeutic effects of hemodiafiltration were analyzed. Case 1: a middle-aged woman was switched from predilution online hemodiafiltration to hemodialysis, following which the α_1_-microglobulin removal rate decreased from 39.1 to 29.9%. A month later, the severe restless legs syndrome occurred. The treatment was then switched to high-efficiency hemodiafiltration and 2 weeks later, these symptoms were resolved. The α_1_-microglobulin removal rate increased to 41.9%. Her symptoms recurred 5 years later with severity; thus, the hemodiafiltration treatment conditions were changed. Under revised conditions, the α_1_-microglobulin removal rate was 42.6%, and her symptoms were alleviated. Continuation of high-efficiency hemodiafiltration led to the resolution of the syndrome at 1 month after recurrence. Case 2: a middle-aged man on hemodialysis developed the restless legs syndrome in the second year of treatment. The α_1_-microglobulin removal rate was 23.8%. After switching to a month-long high-efficiency hemodiafiltration with a removal rate of ≥ 40%, his symptoms were resolved. However, the syndrome recurred after a year with severity. The symptoms were alleviated using various measures. The hemodiafilters were changed, and hemodiafiltration with an α_1_-microglobulin removal rate of ≥ 40% was continued; 2 months later, his symptoms resolved. High-efficiency online hemodiafiltration is an effective therapeutic strategy for restless legs syndrome in dialysis patients. We found, for the first time, that target removal efficiency is an α_1_-microglobulin removal rate of 40% or higher.

**Electronic supplementary material:**

The online version of this article (10.1007/s10047-020-01164-1) contains supplementary material, which is available to authorized users.

## Introduction

Restless legs syndrome (RLS) is mainly divided into primary (idiopathic) and secondary (symptomatic) RLS. The development of primary RLS has not been fully elucidated; however, a dopaminergic neuron hypofunction, the relationship between iron metabolism and dopamine, and genetic predisposition are considered as risk factors for RLS. Secondary RLS is considered to be caused by other diseases or drugs. In particular, the incidence rate is high in pregnant women, patients with iron deficiency anemia, patients with diabetes mellitus and patients with end stage renal disease [1, 2]. The causative substances of RLS in dialysis patients have not been elucidated yet, and no detailed investigation has been performed.

We previously reported that the RLS symptoms in dialysis patients improved through the use of online hemodiafiltration (OL-HDF), which increased the removal efficiency of the low molecular weight proteins (LMWP) [3].

In this study, we aimed to describe the clinical course of two patients with recurrent RLS and investigate the therapeutic effect of high-efficiency OL-HDF.

## Case presentation

Case 1: a female patient in her 50s with polycystic kidney disease (primary disease) and a 12-year history of dialysis at the time of recurrence.

Her condition was stable during predilution online HDF (pre-HDF) using FDY-180GW (PEPA membrane, Nikkiso Co., Ltd). Although OL-HDF was approved by the health insurance (in 2010, indicated for dialysis amyloidosis and disdialysis syndrome), it was not indicated for the pathological condition of this patient. Thus, the method was changed to hemodialysis (HD). The removal rates of β_2_-microglobulin (MG) and α_1_-MG were 83.1% and 82.0%, respectively, for HDF and 39.1% and 29.9%, respectively, for HD. While there was a slight change in the β_2_-MG removal rate following the switch to HD, the α_1_-MG removal rate decreased by 9 points. However, Kt/V for urea increased from 1.8 to 2.3. RLS developed approximately 1 month after the switch from OL-HDF to HD. Symptoms were very severe, with an international restless legs syndrome study group severity scale (IRLS) score of 32 [4].

**Fig. 1:**
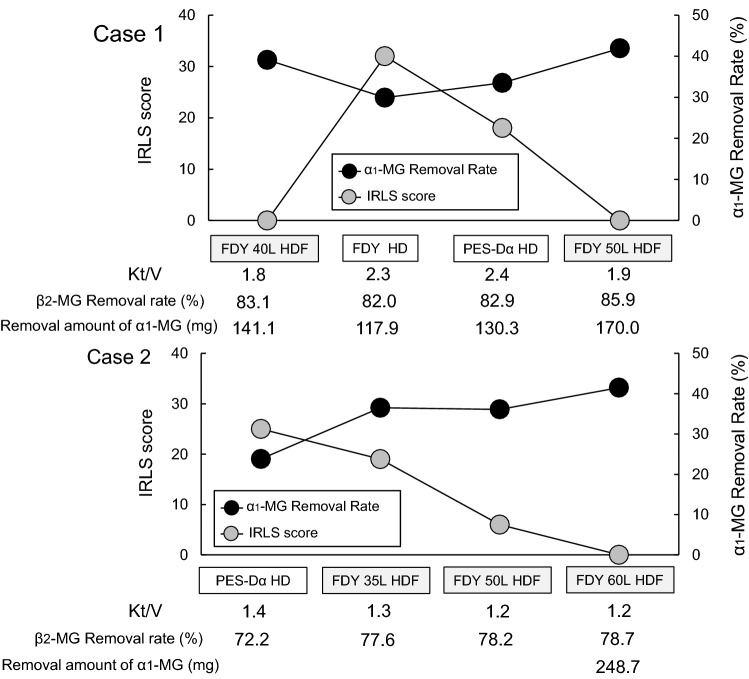
The figure shows the initial onset and the course of RLS in cases 1 and 2. The patient in case 1 was slightly nervous, but the dialysis course was stable with HDF. However, RLS developed 1 month after switching from HDF to HD. The dialysis efficiency of HD was increased, and though RLS was alleviated, it was not resolved. Thus, the method was switched to HDF. In Case 2, RLS developed in the 2nd year of dialysis and, thus, primary RLS was suspected. However, high-efficiency HDF was performed and the patient recovered after 1 month of the entire course

The patient was switched to a high-performance dialyzer and continued to receive longer HD sessions (from 4 to 4.5 h) following which, the RLS symptoms gradually subsided. However, the patient was not completely cured and complained of shoulder joint pain and severe insomnia. Therefore, the dialysis method was changed to 50 L/session(s) pre-HDF; RLS resolved 2 weeks after the change. At the time of RLS resolution, the removal rates of β_2_-MG and α_1_-MG were 85.9% and 41.9%, respectively, (Fig. 1). Changes in the α_1_-MG removal rate (39.1% → 29.9% → 41.9%) correlated with the symptoms during this episode (stabilization → worsening → alleviation). Subsequently, high-efficiency pre-HDF (β_2_-MG removal rate ≥ 80%, α_1_-MG removal rate ≥ 35%) was continued.

RLS recurred in this patient 5 years later. Since the symptoms were severe with an IRLS score of 28, the total substitution fluid (total Qs) for the pre-HDF (blood flow rate [Qb]: 250 mL/min) was increased from 60 to 65 L/s using MFX-19U (PES membrane, Nipro). The treatment time was extended from 4 to 4.5 h. In the third efficiency evaluation after the change in HDF parameters, the removal rates of β_2_-MG and α_1_-MG were found to be 85.9% and 42.6%, respectively, and the IRLS score improved to 21 on the 9th day after recurrence. However, RLS symptoms persisted, and the method was switched back to 50 L/s pre-HDF using GDF-21 (PEPA membrane, Nikkiso Co.,Ltd) for further high-efficiency HDF. The removal rate of α_1_-MG was 48.4%, its removal amount was 190 mg, and the IRLS score improved to 10.

**Fig. 2:**
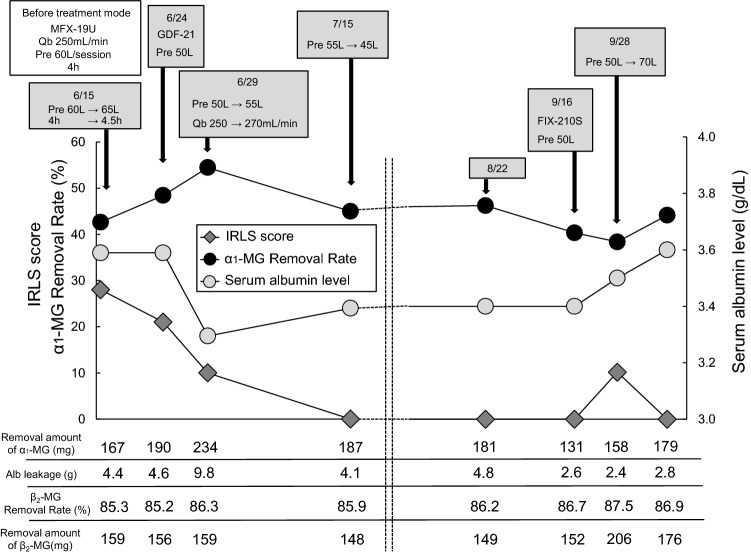
The figure shows the course at the time of recurrence in case 1. There was no apparent decrease in dialysis efficiency, but RLS recurred. After the recurrence, HDF was performed with an α_1_-MG removal rate of ≥ 40%. Alb leakage increased and the serum Alb level decreased, but RLS was resolved. The removal rate and amount of α_1_-MG at the time of RLS resolution after the initial onset were 41.9% and 170 mg, respectively, and after recurrence were 54.5% and 234 mg, respectively. This indicates that higher-efficiency HDF was required at the time of recurrence

RLS was resolved 1 month after recurrence by continuing pre-HDF with the α_1_-MG removal rate of ≥ 45%. The serum albumin (Alb) level was 3.6 g/dL before recurrence, but decreased to 3.3 g/dL with an increase in the amount of Alb leakage. Therefore, after the RLS symptoms disappeared, the total Qs was decreased to 45 L/s. Since the recovery of serum Alb level was delayed, 1 month after the patient was cured, the condition was changed to 50 L/s pre-HDF using FIX-210Seco (ATA membrane, Nipro). Under these conditions, the removal rate of α_1_-MG was 38.0% with a removal amount of 144 mg, and the amount of Alb leakage was 2.6 g/dL. The serum Alb level increased to 3.6 g/dL 2 weeks after switching to FIX. RLS recurred with mild symptoms (IRLS score 5). When the total Qs was increased to 70 L/s, the removal rate of α_1_-MG became 39% with a removal amount of 158 mg. RLS symptoms disappeared 10 days later (Fig. 2).

Case 2: the patient was a man in his 40 s, with a 4-year history of dialysis at the time of recurrence. While the patient was stable on HD, the first episode of RLS occurred 2 years after the start of dialysis. At the onset of RLS, the removal rates of β_2_-MG and α_1_-MG were 72.2% and 23.8%, respectively. The IRLS score improved from 25 to 6 after switching to pre-HDF using FDY-GW with an α_1_-MG removal rate of 36%. Since the symptoms persisted, the total Qs was increased from 50 to 60 L/s. An α_1_-MG removal rate of ≥ 40% was achieved and the HDF continued. RLS resolved after 1 month of the entire course (Fig. 1).

**Fig. 3:**
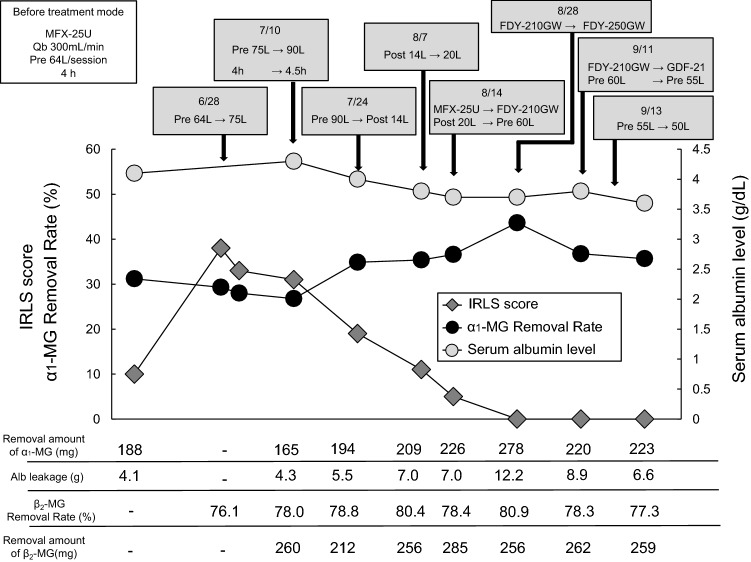
Course at the time of recurrence in case 2. It is strongly suspected that recurrent RLS was caused by deterioration of the hemodiafilter, MFX-U. Even when the total Qs was increased or when post-HDF was performed, the removal rate of α_1_-MG did not reach 40%, which caused a delay in RLS resolution. The patient was successfully treated by FDY-GW with a large pore size. In this patient, the removal rate and amount of α_1_-MG at the time of RLS healing after the initial onset were 41.5% and 248 mg, respectively, and after recurrence were 43.6% and 278 mg, respectively

Following RLS resolution, 64 L/s pre-HDF (Qb: 250 mL/min, treatment time: 4 h) was continued using MFX-25U. The patient sometimes complained of a mild abnormal sensation in the lower extremities; RLS recurred 1 year after healing. The symptoms were very severe with an IRLS score of 38. The performance of MFX-U deteriorated, and the removal rates of β_2_-MG and α_1_-MG were 76% and 29%, respectively, in the pre-HDF, when the total Qs was 64 L/s and Qb was 300 mL/min. The treatment was strengthened by increasing the total Qs and extending the treatment time, without changing the hemodiafilter; however, an α_1_-MG removal rate of ≥  35% could not be obtained. Thus, the method was changed to postdilution online HDF (post-HDF) with a total Qs of 20 L/s; the α_1_-MG removal rate was 35%. Although the IRLS score improved to 5, the RLS was not resolved. Thus, the treatment was switched to pre-HDF using FDY-GW, with the total Qs of 60 L/s. An α_1_-MG removal rate of ≥ 40% was achieved. Although the amount of Alb leakage was excessive, this HDF treatment was continued and RLS resolved approximately 2 months after the recurrence (Fig. 3).

RLS recurred the following year (IRLS score 20) after two conditions of low-efficiency HDF were implemented (the removal rate of α_1_-MG: 1st 25%, 2nd 26%). The treatment was thus revised to 50 L/s pre-HDF (using GDF-21, Qb: 300 mL/min) and 4.5 h of treatment time; RLS resolved 3 weeks after the recurrence. Under these conditions, the removal rates of β_2_-MG and α_1_-MG were 83.1% and 41.4%, respectively, and the amount of Alb leakage was 4.2 g.

During the observation period, no obvious iron deficiency was observed in both patients.

Informed consent for publication of this report was obtained from both patients included in this study.

## Discussion

The International Restless Legs Syndrome Study Group provides diagnostic criteria for RLS, and the criteria for severity classification have also been established [4, 5]. The severity is classified into four grades as follows: mild (score, 0–10), moderate (score, 11–20), severe (score, 21–30), and very severe (score, 31–40).

Previous studies showed that RLS occurred in 12% to 62% of dialysis patients [6–9], and higher incidence rates of RLS among dialysis patients have been reported in studies outside Japan. This may be attributable to the fact that for dialysis therapy in Japan, ultra-pure dialysis fluids and high- or super high-flux dialyzers with excellent biocompatibility are frequently used, which ensure the quality of dialysis to be satisfactory. At our clinic, we encountered nine patients with RLS over the past 10 years (2010–2019). One patient was transferred to another facility, and the clinical course is thus unknown. In the other eight patients, RLS was resolved by high-efficiency HDF. At the time of writing this report (December 2019), 119 patients were receiving maintenance dialysis at our clinic, none of whom had RLS. In addition, none of the patients experienced a new onset of RLS during the last 5 years.

According to our clinical experience, RLS was observed among patients receiving maintenance HD, patients in whom OL-HDF therapy was switched to conventional HD therapy, and patients in whom α_1_-MG removal efficiency decreased due to some causes even during OL-HDF therapy.

At the time of recurrence of RLS in case 1, the manufacturer of the hemodiafilter was requested to explain whether the performance level of the product would decrease at the time of shipment of the hemodiafilter of that particular lot. The person in charge replied that the pore size of the dialysis membrane was within the reference range, although it was at the minimum acceptable limit. Accepting their reply, we took this into consideration regarding the cause of the onset of RLS; however, the removal efficiency of OL-HDF immediately before RLS recurrence in this patient was not evaluated at our clinic, so that the cause of the RLS recurrence remains unknown.

**Fig. 4:**
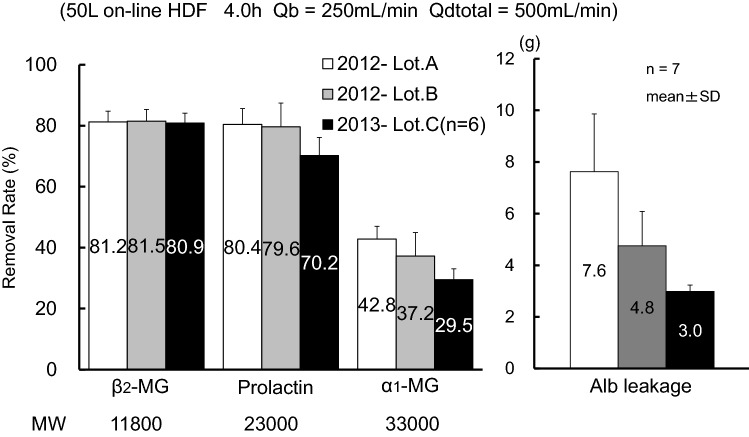
The same product from the same brand may differ in efficiency depending on the production date. To detect this difference, α_1_-MG removal rate and Alb leakage should be measured. If the evaluation is only for β_2_-MG, all products would show the same efficiency

We infer that the RLS recurrence in case 2 was caused by the decreased performance level of the hemodiafilter. High-efficiency hemodiafilters with decreased removal performance levels might be supplied, causing a change in the patient’s clinical response. According to the lot of high-efficiency hemodiafilters, the removal performance levels slightly differed for β_2_-MG and greatly differed for α_1_-MG (Fig. 4).

While the cause of RLS in dialysis patients has not been established, some types of uremic toxin with a molecular weight greater than that of β_2_-MG or protein-bound toxin are considered causative substances [10]. We consider that the accumulation of some types of uremic toxins may cause functional abnormalities (or inflammation) of peripheral nerves, thereby causing RLS. In addition, α_1_-MG has been reported to have an antioxidant property [11–13]. If it is assumed that RLS is caused by chronic inflammation of peripheral nerves due to oxidative stress, the significance of efficient removal of α_1_-MG can be understood. High efficient removal of α_1_-MG promotes the turnover of α_1_-MG by removing α_1_-MG which has lost its antioxidant property and promoting the generation of antioxidant α_1_-MG in the liver. This mechanism may lead to recovery from RLS. Higuchi et al. reported that blood levels of 8-OHdG, an oxidative stress marker, were higher in dialysis patients with RLS than in dialysis patients without RLS, and that blood levels of 8-OHdG correlated with the severity of RLS. The authors suggested an involvement of oxidative stress in the pathogenesis of RLS in dialysis patients [9].

From the above-mentioned perspective, high-efficient removal of α_1_-MG is essential for the treatment of RLS in dialysis patients for the following reasons: (1) direct removal of causative substances categorized in larger LMWPs; (2) increased removal of protein-bound toxins by increasing the amount of Alb leakage; and (3) removal of old non-antioxidant α_1_-MG to increase synthesis of new α_1_-MG in the liver.

For dialysis patients with RLS, aggressive treatment with constant high-efficiency HDF that can achieve an α_1_-MG removal rate of at least 40% should be performed after assessment for iron deficiency. Although excessive Alb leakage cannot be avoided in high-efficiency HDF, the amount of Alb leakage of up to approximately 8–9 g should be set as an acceptable level in the early phase of treatment to promptly improve the patient’s quality of life. In case 2 in this report, in addition to the increase in α_1_-MG removal rate, the protein-bound toxins were successfully removed by setting the amount of Alb leakage to 5.5 g/s or more, which may have resulted in the cure of RLS.

Specific conditions are as follows: treatment with pre-HDF using GDF, MFX-U, or FIX-U should be started with a Qb of 250–300 mL/min, total Qs of 50 L/s, and treatment time of 4 h. Treatment with post-HDF using FIX-S, ABH-PA, or NVF-H should be started with a Qb of 250–300 mL/min, total Qs of 12 L/s, and treatment time of 4 h. During HDF therapy, therapeutic effects should be confirmed by examinations for α_1_-MG removal rates and amounts, and amounts of Alb leakage and high-efficiency HDF should be continued under appropriate treatment conditions. Extension of treatment time contributes to shortening of treatment duration.

If the therapeutic effect is not improved even with high-efficiency HDF, an excessively higher removal rate of α_1_-MG, as obtained by hematocrit correction, may be indicated. The removal amount of α_1_-MG should be examined in consideration of any dissociation between its removal rate and removal amount. This can occur when plasma refilling is slow or when blood recirculates within the blood circuit. In such cases, the treatment conditions of HDF should be set with a target α_1_-MG removal amount of 180 mg or, when possible, 200 mg.

With the same α_1_-MG removal efficiency, no superiority or inferiority in clinical effects was observed between the two treatment modes of pre- and post-HDF. We consider that OL-HDF should be continued for 1 month after RLS resolution under the dialysis conditions at the time of resolution.

## Conclusion

Two cases of recurrent RLS in dialysis patients are presented in this study. High-efficiency online HDF is an effective therapeutic strategy for severe or very severe RLS (IRLS score: 21 or more) in dialysis patients, having reached the target removal efficiency in terms of α_1_-MG removal rate of 40% or higher and α_1_-MG removal amount of 200 mg.

## Electronic supplementary material

Below is the link to the electronic supplementary material.

Supplementary file1 (PDF 155 kb)
