# Supplementary material for: Therapeutic effect of high-efficiency online hemodiafiltration for recurrent restless legs syndrome in dialysis patients
Source: J Artif Organs. 2020 Mar 30;23(3):296–301. doi: 10.1007/s10047-020-01164-1 (PMC7458888; doi:10.1007/s10047-020-01164-1)
Supplement: Supplementary file 1 — Supplementary file1 (PDF 155 kb) [file 10047_2020_1164_MOESM1_ESM.pdf]

Supplementary Figures

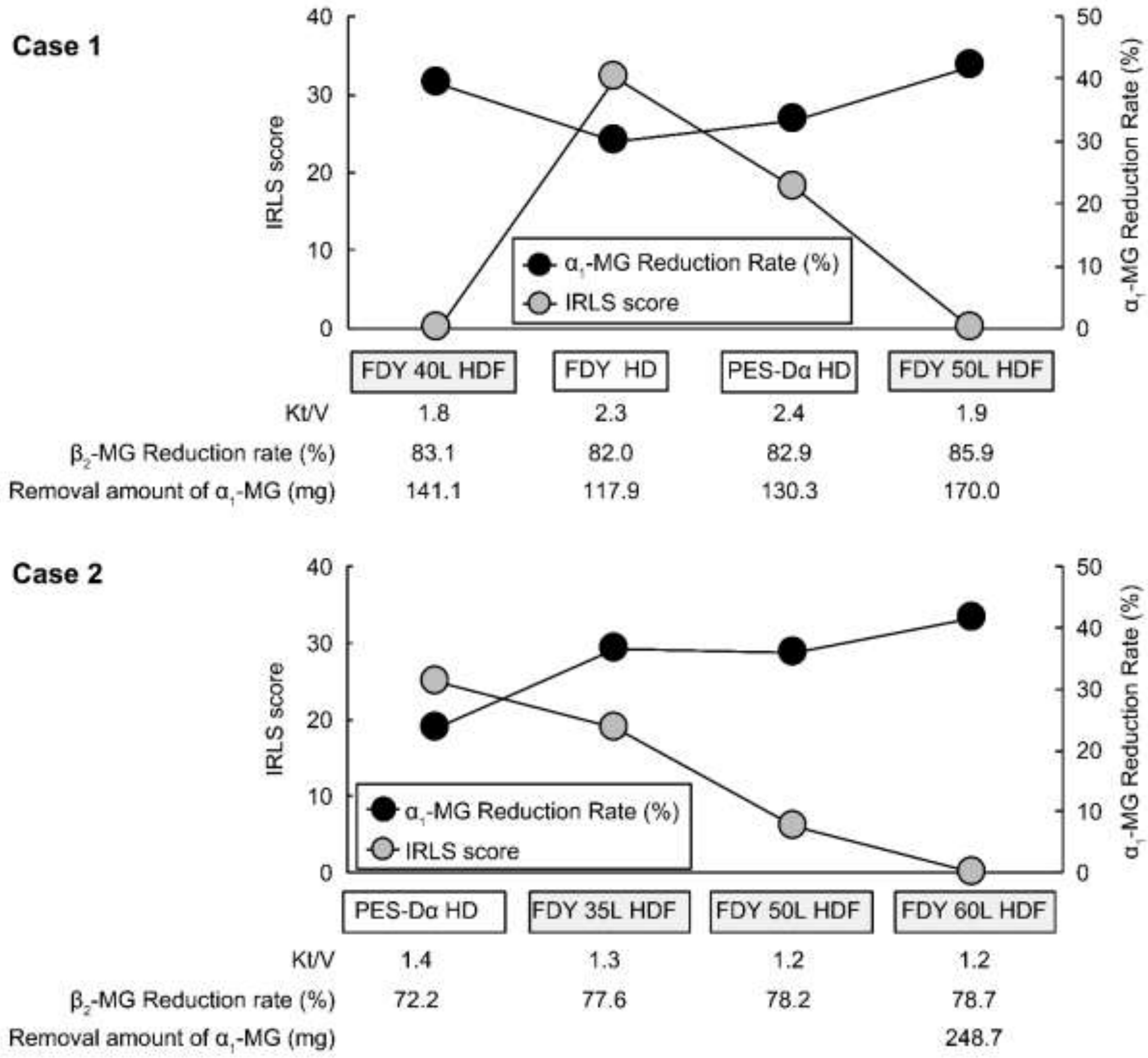

Supplementary Fig. S1 The figure shows the initial onset and the course of RLS in Cases 1 and 2. The patient in Case 1 was slightly nervous, but the dialysis course was stable with HDF. However, RLS developed 1 month after switching from HDF to HD. The dialysis efficiency of HD was increased, and though RLS was alleviated, it was not resolved. Thus, the method was switched to HDF. In Case 2, RLS developed in the 2nd year of dialysis and, thus, primary RLS was suspected. However, high-efficiency HDF was performed and the patient recovered after 1 month of the entire course

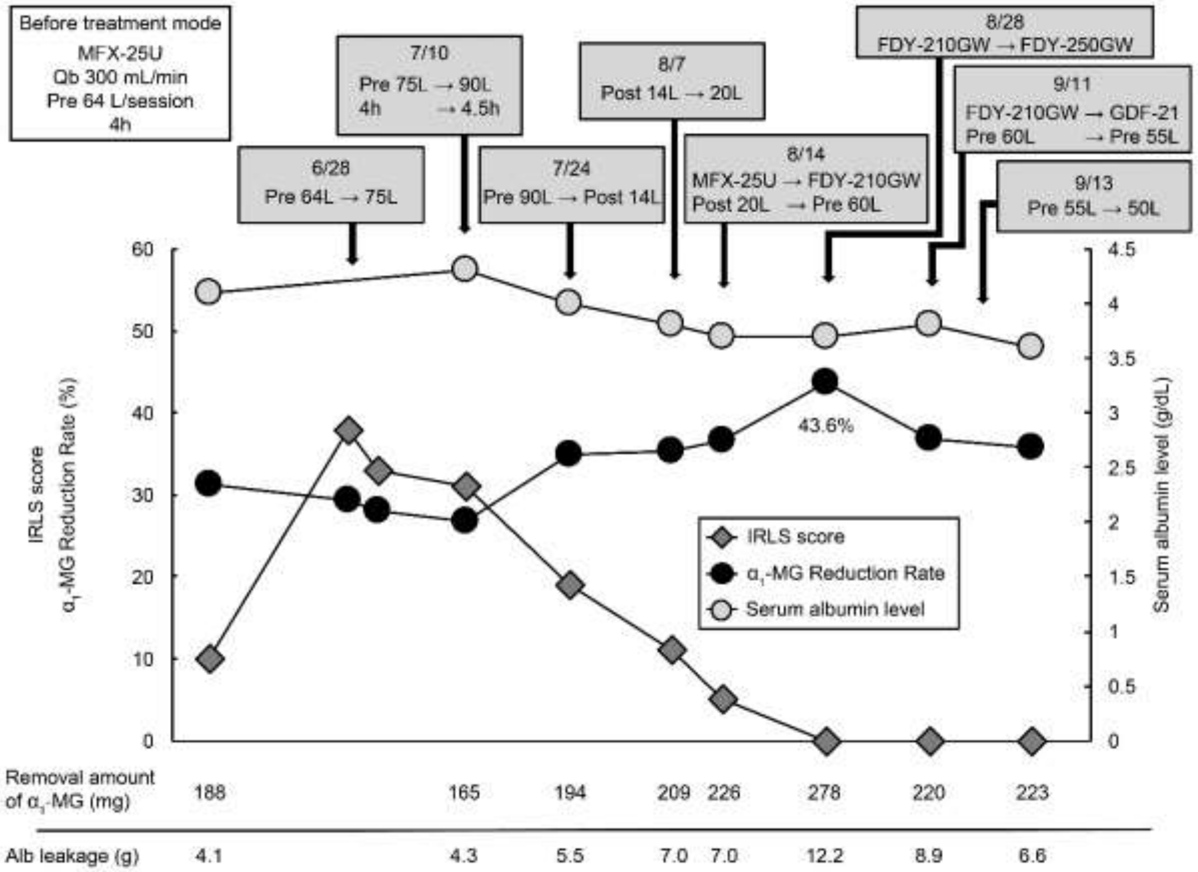

Supplementary Fig. S2 Course at the time of recurrence in Case 2. It is strongly suspected that recurrent RLS was caused by deterioration of the hemodiafiltration MFX-U. Even when the total replacement solution was increased or when postdilution HDF was performed, the removal rate of α1-MG did not reach 40%, which caused a delay in RLS resolution. The patient was successfully treated by using FDY-GW with a large pore size. In this patient, the removal rate and amount of α1-MG at the time of RLS healing after the initial onset were 41.5% and 248 mg, respectively, and after recurrence were 43.6% and 278 mg, respectively

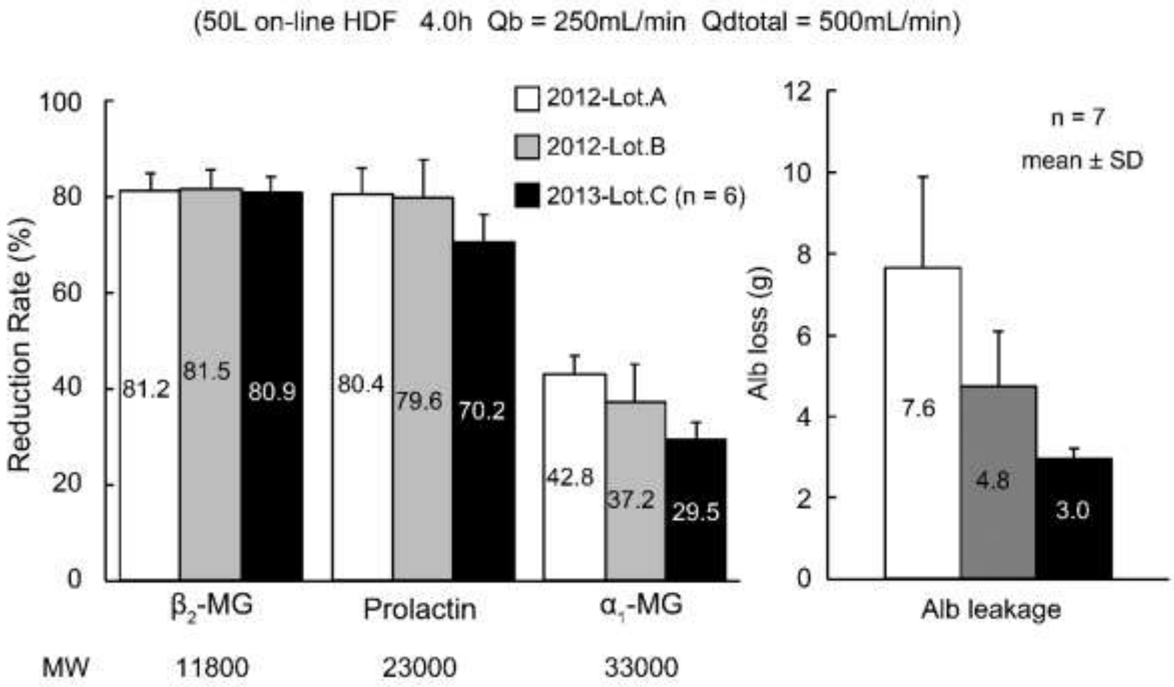

Supplementary Fig. S3 The same product from the same brand may differ in efficiency depending on the production date. To detect this difference, α1-MG removal rate and Alb leakage should be measured. If the evaluation is only for β2-MG, all products would show the same efficiency
